# Phylogenetic Assessment of Understudied Families in *Hymenochaetales* (*Basidiomycota*, *Fungi*)—Reporting Uncovered Species and Reflecting the Recent Taxonomic Updates in the Republic of Korea

**DOI:** 10.1007/s12275-024-00120-5

**Published:** 2024-05-16

**Authors:** Yoonhee Cho, Dohye Kim, Young Woon Lim

**Affiliations:** School of Biological Sciences and Institute of Microbiology, Seoul National University, Seoul, 08826 Republic of Korea

**Keywords:** *Hirschioporaceae*, *Rickenella*, *Rickenellaceae*, *Rigidoporaceae*, *Sideraceae*, *Skvortzoviaceae*, *Trichaptum*, *Tubulicrinaceae*

## Abstract

*Hymenochaetales* Oberw. is an order classified in *Basidiomycota* of *Fungi*, and species in this order display notable diversity. They exhibit various fruiting body shapes, including clavarioid, effused-reflexed, and resupinate basidiomes. Few mycorrhizal species have been reported in *Hymenochaetales*, but wood-decaying species dominate the order. *Hymenochaetaceae* Imazeki & Toki and *Schizoporaceae* Jülich are the most species-rich families within *Hymenochaetales*, and most species in the Republic of Korea belong to these two families. As such, current taxonomic classification and nomenclature are not reflected upon species in the remaining *Hymenochaetales* families. For this study, a multifaceted morphological and multigenetic marker-based phylogenetic investigation was conducted to, firstly, comprehensively identify understudied *Hymenochaetales* specimens in Korea and, secondly, reflect the updates on the species classification. Five genetic markers were assessed for the phylogenetic analysis: nuclear small subunit ribosomal DNA (nSSU), internal transcribed spacer (ITS), nuclear large subunit ribosomal DNA (nLSU), RNA polymerase II subunit 2 gene (*RPB2*), and translation elongation factor 1 gene (*TEF1*). The results from phylogenetic analysis supported 18 species classified under eight families (excluding *Hymenochaetaceae* and *Schizoporaceae*) in Korea. Species formerly placed in *Rickenellaceae* and *Trichaptum *sensu lato have been systematically revised based on recent taxonomic reconstructions. In addition, our findings revealed one new species, *Rickenella umbelliformis*, and identified five formerly nationally unreported species classified under five understudied families. Our findings contribute to a better understanding of *Hymenochaetales* diversity and highlight the need for continued research.

**Supplementary Information:**

The online version contains supplementary material available at 10.1007/s12275-024-00120-5.

## Introduction

*Hymenochaetales* Oberw. was once considered a group of fungi causing white rot, bearing setae, and having an imperforate parenthosome and no clamps (Hibbett & Thorn, 2001). However, multiple taxonomic revisions based on nuclear large subunit ribosomal DNA (nLSU) phylogeny (Larsson, 2007; Redhead et al., 2002) have introduced external basidiomycetes into *Hymenochaetales* that cumulated exceptions to these commonalities (van Driel et al., 2009). Now, the order includes mycorrhizal species, such as *Coltricia* species (Tedersoo et al., 2007), and species of diverse morphological characters. Various basidiome types are found across the order, such as clavarioid, effused-reflexed, resupinate, stipitate stereoid, and stipitate lamellate basidiomes (Dentinger & McLaughlin, 2006; Larsson et al., 2006). Collectively, 69 *Hymenochaetales* genera have been reported up to date (Wang et al., 2023; Zhou et al., 2023), with species numbers growing rapidly since the last estimate of 600 in 2008 (Kirk et al., 2008).

Most *Hymenochaetales* species are classified under two well-known families, *Hymenochaetaceae* Imazeki & Toki and *Schizoporaceae* Jülich, from 16 accepted families. Many families of the remaining 14 are small in species numbers, several being monogeneric. Most have been recently supported at a family level based on a concatenated dataset of multiple genetic markers, including nuclear small subunit ribosomal DNA (nSSU), internal transcribed spacer (ITS), nLSU, mitoribosomal small subunit (mt-SSU), translation elongation factor 1 gene (*TEF1*), RNA polymerase II subunit 1 gene (*RPB1*), and RNA polymerase II subunit 2 gene (*RPB2*) (Wang et al., 2023; Zhou et al., 2023). Compared to the traditional use of ITS and nLSU markers, multiple genetic markers have significantly increased the resolution of taxon delimitation in *Hymenochaetales*, revealing numerous new species and allowing better species identification (Cho et al., 2023; Liu et al., 2021).

Reports of indigenous *Hymenochaetales* species in the Republic of Korea were also concentrated within *Hymenochaetaceae* and *Schizoporaceae* (Jang et al., 2016; Kim et al., 2009, 2016). The incorporation of molecular data in evaluating specimens from *Hymenochaetaceae* and *Schizoporaceae* has expanded the documentation of species previously unreported within the country (Cho et al., 2021; Kim et al., 2016), and this has contributed to a more comprehensive account of species diversity in these families. For the others, there were only two families (*Rickenellaceae* Vizzini and *Tubulicrinaceae* Jülich), six genera, and fourteen species registered in the national list (https://species.nibr.go.kr/; accessed 2023-11-15), where most were identified based on macromophological characters and/or a single genetic marker (Kim et al., 1976; Park et al., 2017). A more profound research of these families based on multigenetic marker phylogeny would accurately identify or verify these species and unveil the true diversity of *Hymenochaetales* in Korea.

The main objectives of the current study were to (i) identify species of understudied *Hymenochaetales* families in Korea and (ii) reflect the latest systematic classification for these species. To achieve these two objectives, we conducted a multifaceted morphological observation and multigenetic marker-based phylogenetic analysis. Five genetic markers were analyzed for phylogeny, namely nSSU, ITS, nLSU, *RPB2*, and *TEF1*. These regions had been selected based on the ease of amplification, thus acquisition of sequences, and the availability of reference sequences in the public database. As a result, one new species was confirmed and described, and the genus *Sidera* and five species, each in a different family, were revealed for the first time in Korea.

## Materials and Methods

### Study Materials

*Hymenochaetales* specimens collected from Korea were stored as dry specimens at the Seoul National University Fungus Collection (SFC). Twenty-one specimens classified under understudied *Hymenochaetales* families (excluding *Hymenochaetaceae* and *Schizoporaceae*) were selected for the present study based on preliminary ITS-based species identification (methods described below). The selected specimens were collected over the period of 2014 to 2023. Field photographs and information on the collection date, location, and additional notes of fresh fruiting bodies were available for each specimen.

### DNA Extraction, PCR, and Sequencing

Fungal tissue of approximately 1 × 1 cm was isolated from each specimen for genomic DNA extraction. Tissue grinding was done using a Bead Ruptor Elite (OMNI International) in 200 μl of 2 × Cetyltrimethyl ammonium bromide (CTAB) buffer. The remaining steps were conducted using the AccuPrep Genomic DNA Extraction Kit (Bioneer).

For the polymerase chain reaction (PCR), a PCR reaction mixture was prepared using a PCR Premix (Bioneer). For each reaction, 1–2 µl of DNA was used. PCR was performed using a C1000 thermal cycler (Bio-Rad). The ITS region was amplified with ITS1F and ITS4B primers (Gardes & Bruns, 1993) under the following conditions: 95℃ for 5 min; 35 cycles of 95℃ for 40 s, 55℃ for 40 s, and 72℃ for 1 min; and 72℃ for 5 min. The nLSU region was amplified with LR0R and LR5/LR7 primers (Vilgalys & Hester, 1990) under the following conditions: 95℃ for 5 min; 35 cycles of 95℃ for 40 s, 55℃ for 40 s, and 72℃ for 1.5 min; and 72℃ for 5 min. The nSSU region was amplified with NS1 and NS4 primers (White et al., 1990) under the same condition for nLSU. The *RPB2* region was amplified with fRPB2-5F and fRPB2-7.1R primers (Matheny, 2005) under the following conditions: 95℃ for 5 min; 10 cycles of 95℃ for 40 s, 60℃ for 40 s, and 72℃ for 2 min; 35 cycles of 95℃ for 40 s, 55℃ for 1.5 min, 72℃ for 2 min; and 72℃ for 10 min. The *TEF1* region was amplified with EF1-983F and EF1-1567R primers (Rehner & Buckley, 2005) under the following conditions: 95℃ for 5 min; 35 cycles of 95℃ for 40 s, 59℃ for 40 s, and 72℃ for 1.5 min; and 72℃ for 5 min.

All PCR products were verified by gel electrophoresis using a 1% agarose gel and Gel Doc™ XR (Bio-Rad). The PCR products were purified using the Expin™ PCR Purification Kit (GeneAll Biotechnology). DNA sequencing was performed with the PCR primers at Bioneer using an ABI Prism 3730XL machine (Applied Biosystems). Preliminary identification for sequences was performed through NCBI BLAST (using the blastn database) to eliminate contaminated data and select specimens of understudied *Hymenochaetales* families based on ITS. After a manual quality check for chimeras, mixed peaks, and noise, the forward and reverse sequence reads were assembled using Geneious Prime 2023.0.3 (http://www.geneious.com/). The final sequences are deposited in GenBank (Table 1).

**Table 1:** Species, strains, and GenBank accessions for sequences used in multigenetic marker-based phylogeny

Species	Strain	GenBank Accessions				
		nSSU	ITS	nLSU	*RPB2*	*TEF1*
*Alloclavaria purpurea*	H6047663	MF318995	MF319055	MF318905		
*Alloclavaria purpurea*	M. Korhonen 10305	MF318986	MF319044	MF318895		
*Atheloderma mirabile*	TAA 169235		DQ873592	DQ873592		
*Basidioradulum radula*	LWZ 20201017-62	ON063814	ON063684	ON063884	ON100713	ON089691
*Blasiphalia pseudogrisella*	P. Hoijer 4118	MF318989	MF319047	MF318898		
*Blasiphalia pseudogrisella*	P. Hoijer 4393	MF318990	MF319048	MF318899		
*Bridgeoporus sinensis*	Cui 10013		KY131832	KY131891		
*Bryopistillaria sagittiformis*	IO.14.164		MT232349	MT232303	MT242333	
*Cantharellopsis prescotii*	H6059300	MF318993	MF319051	MF318903	MF288855	
*Coltricia abieticola*	Cui 10321	KY693761	KX364785	KX364804	KX364876	KY693911
*Coniferiporia weirii*	FP-134599-SP (Dai 22935)		MT420695	MT416461	MT386025	MT470379
*Contumyces rosellus*	MGW1462	MF319001	MF319059	MF318912	MF288859	
*Contumyces vesuvianus*	203608	MF319002		MF318913	MF288860	
*Cotylidia fibrae*	**FM639**		**NR_176148**	**NG_088193**		
*Cotylidia* sp.	AFTOL-700	AY705958	AY854079	AY629317	AY883422	AY885148
*Fasciodontia yunnanensis*	LWZ 20190811-50a	ON063804	ON063675		ON100704	
*Fomitopsis pinicola*	AFTOL 770	AY705967	AY854083	AY684164	AY786056	AY885152
*Ginnsia viticola*	Wu 0010-29		MN123802	GQ470670		
*Globulicium hiemale*	Hjm 19007		DQ873595	DQ873595		
*Gyroflexus brevibasidiata*	IO.14.230		MT232351	MT232305		
*Hastodontia halonata*	HHB-17058		MK575207	MK598738		
*Heterobasidion annosum*	06129/6		KJ583211	KJ583225	KF006499	KX252741
*Hirschioporus abietinus*	Cui 2667	OQ449408	OQ449096	OQ449033		OQ831431
*Hirschioporus abietinus*	Dai 23760	OQ449410	OQ449039	OQ449034		
*Hirschioporus abietinus*	KUC20130719-05		KJ668437	KJ668289		
*Hirschioporus acontextus*	Dai 19097	OQ449012	OQ449140	OQ449199		
*Hirschioporus acontextus*	**Dai 23793**	**OQ449013**	**OQ449141**	**OQ449200**		**OQ831439**
*Hirschioporus acontextus*	KUC20131001-03		KJ668436	KJ668288		
*Hirschioporus beijingensis*	Dai 18907	OQ449014	OQ449142	OQ449201		OQ831440
*Hirschioporus beijingensis*	**Dai 23704**	**OQ449015**	**OQ449143**	**OQ449202**		**OQ831441**
*Hirschioporus chinensis*	Dai 20264	OQ449017	OQ449101	OQ449204		OQ831443
*Hirschioporus chinensis*	Dai 23048	OQ438032	OQ437349	OQ438002		OQ831445
*Hirschioporus fuscoviolaceus*	Cui 10439	OQ438041	OQ437361	OQ438010		OQ831452
*Hirschioporus fuscoviolaceus*	Dai 20988	OQ438039	OQ437357	OQ438006		OQ831449
*Hirschioporus pubescens*	**Dai 17064**	**OQ449152**	**OQ437377**	**OQ438019**		
*Hirschioporus pubescens*	Dai 23710	OQ449153	OQ512026	OQ449059		OQ857930
*Hirschioporus pubescens*	KA17-0228		MN294864			
*Hirschioporus tianschanicus*	Dai 19064	OQ449161	OQ437386	OQ449066		OQ857946
*Hirschioporus tianschanicus*	**Dai 19067**	**OQ449162**	**OQ448960**	**OQ449067**		**OQ857947**
*Hydnoporia tabacina*	LWZ 20210924-26a	ON063778	ON063651	ON063851	ON100685	ON089676
*Hyphodontia densispora*	LWZ 20170908-5		MT319426	MT319160		
*Hyphodontia zhixiangii*	LWZ 20170818-13		MT319420	MT319151	MT326270	MT326397
*Kneiffiella barba-jovis*	KHL 11730		DQ873609	DQ873610		
*Kneiffiella eucalypticola*	LWZ 20180509-11		MT319410	MT319142		
*Kneiffiella subglobosa*	LWZ 20180416-6		MT319413	MT319145		
*Lawrynomyces capitatus*	KHL 8464		DQ677491	DQ677491		
*Leifia brevispora*	LWZ 20170820-48		MK343470	MK343474		
*Leucophellinus hobsonii*	Cui 6468		KT203288	KT203309	KT210365	
*Leucophellinus irpicoides*	Yuan 2690		KT203289	KT203310	KT210366	
*Loreleia marchantiae*	Lutzoni 930826-1		U66432	U66432		
*Lyomyces sambuci*	LWZ 20180905-1	ON063807	MT319444	MT319178	MT326291	MT326391
*Muscinupta laevis*	OTA:70515		OQ064898			
*Muscinupta laevis*	V. Haikonen 19745	MF319004	MF319066	MF318921	MF288861	
*Nigrohirschioporus durus*	Dai 20642	OQ449103	OL470321	OL462835		OQ857954
*Nigrohirschioporus durus*	He 20120724-11	OQ449104	OQ448973	OQ449076		OQ857955
*Nigrohirschioporus griseofuscus*	B3942	OQ449106	OQ448975	OQ438022		OQ857957
*Nigrohirschioporus griseofuscus*	JV 1909/ 6	OQ449107	OQ437343	OQ438024		OQ857958
*Nigrohirschioporus sector*	AS 2707	OQ449108	OQ437344	OQ438025		OQ857959
*Nigrohirschioporus sector*	B3799	OQ449109	OQ437345	OQ438026		OQ857960
*Nigrohirschioporus trimiticus*	B4105	OQ453487	OQ453307	OQ453534		OQ857963
*Nigrohirschioporus trimiticus*	**B696**	**OQ453488**	**OQ453308**	**OQ453535**		
*Onnia tomentosa*	Dai 22935		OL473604	OL473617	OM937016	OM800825
*Pallidohirschioporus biformis*	Dai 12746	OQ453491	OQ453311	OQ453538		OQ874735
*Pallidohirschioporus biformis*	Dai 19466	OQ453501	OQ453223	OQ453548		OQ874743
*Pallidohirschioporus biformis*	KA17-0213		MN294787			
*Pallidohirschioporus biformis*	SFC20170810-02		MT044404			
*Pallidohirschioporus brastagii*	Dai 22919	OQ453251	OQ453371	OQ453297		OQ874751
*Pallidohirschioporus polycystidiatus*	Dai 19100	OQ453255	OQ453378	OQ453301		OQ874757
*Pallidohirschioporus versicolor*	Dai 19331	**OQ453261**	**OQ453386**	**OQ474951**		**OQ874762**
*Peniophorella aspersa*	CLZhao 17063		OM985730	OM985771		
*Peniophorella aspersa*	**F24809**		**NR_172775**	**NG_073750**		
*Peniophorella crystallifera*	**F23666**		**NR_171802**	**NG_073751**		
*Peniophorella crystallifera*	LWZ 20210626-4a	ON063815	ON063685	ON063885		
*Peniophorella fissurata*	**Zhao 9421**		**NR_177497**	**NG_154027**		
*Peniophorella odontiiformis*	CLZhao 9862		MT247004	OM985779		
*Peniophorella odontiiformis*	**MUCL 32673**		**NR_119601**			
*Peniophorella odontiiformis*	SFC20150108-37	OQ996183	OQ996168	OQ996199	OR360738	OR360748
*Peniophorella odontiiformis*	SFC20150113-08	OQ996184	OQ996169	OQ996200	OR360739	OR360749
*Peniophorella odontiiformis*	SFC20160906-22	OQ996185	OQ996170	OQ996201	OR360740	OR360750
*Peniophorella odontiiformis*	SFC20191015-02	OQ996186	OQ996171	OQ996202	OR360741	OR360751
*Peniophorella pallida*	CLZhao 3017		OM985738	OM985780		
*Peniophorella pertenuis*	**FCUG 2430**		**NR_119599**			
*Peniophorella praetermissa*	AFTOL-ID 518	AY707094	AY854081	AY700185	AY787221	AY885150
*Peniophorella praetermissa*	**FCUG 1708**		**NR_119598**			
*Peniophorella praetermissa*	LWZ 20180903-14	ON063816	ON063686	ON063886	ON100714	ON089699
*Peniophorella pubera*	KUC11051		KJ714003			
*Peniophorella pubera*	LWZ 20210624-16b	ON063817	ON063687	ON063887	ON100715	
*Peniophorella pubera*	SFC20180601-02		MK992822			
*Peniophorella reticulata*	**F22559**		**NR_172776**	**NG_073752**		
*Peniophorella rude*	F30649		MN062105	MN062153		
*Peniophorella rude*	LWZ 20171026-7	ON063818	ON063688	ON063888	ON100716	ON089692
*Peniophorella subpraetermissa*	LWZ 20190816-3b	ON063819	ON063689	ON063889	ON100717	
*Peniophorella subpraetermissa*	SFC20180719-01		MK992844			
*Peniophorella subpraetermissa*	**Wu 950627**		**NR_119600**			
*Peniophorella yunnanensis*	**Zhao 6137**		**NR_177498**			
*Perennihirschioporus fumosoavellaneus*	JV 2203/ 80	OQ450176	OQ474940	OQ474955		OQ874768
*Perennihirschioporus variabilis*	B856	OQ450177	OQ474942	OQ474957		OQ874769
*Phellinopsis conchata*	L-7601		KU139188	KU139257	KU139315	KU139377
*Polyporus squamosus*	Cui 10595	KU189840	KU189778	KU189809	KU189988	KU189925
*Repetobasidium conicum*	KHL 12338	DQ873646	DQ873647	DQ873647		
*Resinicium austroasianum*	**LWZ 20180417-5**		**NR_173962**	**NG_088188**		
*Resinicium austroasianum*	LWZ 20191208-11	ON063821	ON063691	ON063891	ON100720	ON089694
*Resinicium bicolor*	AFTOL-810		DQ218310	AF393061	DQ457635	DQ061277
*Resinicium friabile*	LWZ 2021092323a	ON063822	ON063692		ON100719	ON089695
*Resinicium furfuraceum*	FP-101917	DQ834913		DQ863696		
*Resinicium lateastrocystidium*	**LWZ 20180414-15**		**NR_173963**	**MW414455**		
*Resinicium lateastrocystidium*	LWZ 20180416-10		MW414510	MW414456		
*Resinicium monticola*	**FP-150360**		**NR_111226**	**DQ863697**		
*Resinicium mutabile*	**FP-102989**	**NG_064907**	**NR_119612**	**DQ863699**		
*Resinicium rimulosum*	**BPI 878250**		**NR_119611**			
*Resinicium rimulosum*	KUC20131022-12		KJ668464	KJ668315		
*Rickenella danxiashanensis*	GDGM45513	ON063823	MF326424		ON100721	ON089700
*Rickenella fibula*	13109	MF319023		MF318938	MF288876	
*Rickenella fibula*	H6059291	MF319016	MF319091	MF318947	MF288869	
*Rickenella fibula*	HBK012	MF319011	MF319080	MF318940	MF288864	
*Rickenella fibula*	HBK013	MF319012	MF319081	MF318941	MF288865	
*Rickenella fibula*	HBK014	MF319013	MF319082	MF318942	MF288866	
*Rickenella fibula*	HBK015	MF319014	MF319083	MF318943	MF288867	
*Rickenella fibula*	HBK016	MF319015	MF319084	MF318944	MF288868	
*Rickenella fibula*	I. Kytovuori 96-071	MF319025	MF319092	MF318948	MF288870	
*Rickenella fibula*	JMB101914-06	MF319017	MF319086	MF318950	MF288871	
*Rickenella fibula*	JMB101914-07	MF319018	MF319087	MF318951	MF288872	
*Rickenella fibula*	MES950	MF319024	MF319096	MF318957	MF288877	
*Rickenella fibula*	PBM2503	MF319021	DQ241782	MF318953	DQ408115	DQ435794
*Rickenella fibula*	PBM2506	MF319022	MF319093	MF318954	MF288874	
*Rickenella fibula*	RAS051	MF319019	MF319094	MF318972	MF288875	
*Rickenella fibula*	SAT11-173-02	MF319029		MF318955	MF288878	
*Rickenella fibula*	SFC20230704-06	OR758641	OR758634	OR758646	OR819856	OR819858
*Rickenella indica*	**K 190585**		**NR_137857**			
Rickenella indica**	SFC20140626-39	OQ996187	OQ996172	OQ996203		OR360752
*Rickenella mellea*	CBS 579.87		MH862106	MH873795		
*Rickenella mellea*	CBS 581.87		MH862107	MH873796		
*Rickenella minuta*	MES1054	MF319033	MF319102	MF318965	MF288879	
*Rickenella minuta*	MES1535	MF319034	MF319099	MF318958	MF288883	
*Rickenella minuta*	MES1558	MF319038	MF319104	MF318969	MF288885	
*Rickenella minuta*	MES1656	MF319036	MF319103	MF318962	MF288884	
*Rickenella minuta*	MES1781	MF319031	MF319107	MF318959	MF288882	
*Rickenella minuta*	MES1891	MF319037	MF319098	MF318961	MF288887	
*Rickenella minuta*	MES1892	MF319032	MF319108	MF318966	MF288881	
*Rickenella minuta*	MES1950	MF319039	MF319097	MF318964	MF288888	
*Rickenella minuta*	MES1965	MF319040	MF319105	MF318963	MF288886	
*Rickenella swartzii*	HBK017	MF319042	MF319085	MF318976	MF288890	
Rickenella umbelliformis*	**SFC20150701-65**	OQ996188	OQ996173	OQ996204	OR360742	OR360753
Rickenella umbelliformis*	SFC20160512-10	OQ996189	OQ996174			OR360754
Rickenella umbelliformis*	SFC20160713-77	OQ996190	OQ996175	OQ996205	OR360743	OR360755
Rickenella umbelliformis*	SFC20180704-81	OQ996191	OQ996176	OQ996206	OR360744	OR360756
*Rigidoporus corticola*	KUC20130718-79		KJ668502	KJ668354		
*Rigidoporus corticola*	LWZ 20190819-3b	ON063801	ON063673	ON063872		
*Rigidoporus corticola*	SFC20230816-48	OR758642	OR758635	OR758647		
*Rigidoporus cuneatus*	Cui 10855		OQ930254	OQ924530		
*Rigidoporus cuneatus*	Dai 7339		KT203294	KT203315	KT210370	
*Rigidoporus cuneatus*	KA18-0779		MK351747			
*Rigidoporus cuneatus*	LWZ 20190819-5a	ON063802		ON063873	ON100701	
*Rigidoporus ginkgonis*	Cui 5555		KT203295	KT203316	KT210371	
Rigidoporus ginkgonis**	SFC20230630-23	OR758643	OR758636	OR758648	OR819857	
*Rigidoporus juniperinus*	Dai 17100		OQ930261	OQ924537		
*Rigidoporus juniperinus*	YG 1070		MK433641	MK433643		
*Rigidoporus microporus*	JV 2110/1		OQ930266			
*Rigidoporus microporus*	SFC20170119-07		MZ621241			
*Rigidoporus piceicola*	KUC20200924_53		OK559812		ON204049	
*Rigidoporus vinctus*	SFC20170119-34		MZ621227			
*Sanghuangporus weigelae*	LWZ 20210623-2a	ON063799	ON063671	ON063870	ON100697	ON089687
*Schizocorticium lenis*	**LWZ 20180921-7**	**ON063827**	**MW414521**	**MW414467**	**ON100726**	
*Schizocorticium magnosporum*	**Wu 1510-34**		**MK405351**	**MK405337**		**LC567450**
*Schizocorticium mediosporum*	**Chen 2456**		**MK405359**	**MK405345**		**LC567441**
*Schizocorticium parvosporum*	**GC 1508-127**		**MK405361**	**MK405347**		**LC567445**
*Sidera* cf. *vulgaris*	Dai 22822		OM974254	OM974246		
*Sidera inflata*	Cui 13610	MW418088	MW198480			
*Sidera minutipora*	Cui 16720	MW418078	MN621349	MN621348	MW505865	MW446248
*Sidera minutissima*	Dai 19529	MW418079	MN621352	MN621350		
*Sidera parallela*	Dai 22038		MW477793	MW474964		
*Sidera roseo-bubalina*	**BJFC 007251**	**NG_074967**	**NR_172801**			
*Sidera srilankensis*	Dai 19581	MW418089	MN621345	MN621347		MW427604
*Sidera srilankensis*	**Dai 19654**	**NG_073571**	**NR_172780**	**NG_075310**	**MW505868**	**MW427602**
*Sidera tenuis*	**Dai 18697**	**MW418083**	**NR_171833**	**NG_075283**	**MW505866**	**MW427600**
*Sidera tenuis*	Dai 18698	MW418084			MW505867	MW427601
*Sidera tibetica*	**Dai 23648**		**NR_177641**	**OM974245**		
Sidera tibetica**	SFC20230317-17	OQ996192	OQ996177	OQ996207	OR360745	OR360757
*Sidera vesiculosa*	**BJFC 025377**		**NR_164588**	**NG_066418**		
*Sidera vulgaris*	Dai 21057	MW418090	MW198484	MW192009	MW505869	MW427603
*Skvortzovia dabieshanensis*	**LWZ 20201012-22**		**NR_173964**	**NG_088189**		
*Skvortzovia dabieshanensis*	LWZ 20210918-15b	ON063825	ON063694	ON063894	ON100723	ON089696
Skvortzovia dabieshanensis**	SFC20190322-05	OQ996194	OQ996179	OQ996209		
*Skvortzovia pinicola*	LWZ 20210623-18b	ON063826	ON063695	ON063895	ON100724	
*Skvortzovia pinicola*	SFC20151020-15		MF437009			
*Skvortzovia qilianensis*	**LWZ 20180904-16**		**NR_173965**	**NG_088190**		
*Skvortzovia qilianensis*	LWZ 20180904–20	ON063824	MW414520	MW414466	ON100722	
*Skvortzovia yunnanensis*	CLZhao 16084		MW472754	MW473473	ON100725	ON089697
*Tremellodendron pallidum*	AFTOL 699	AY766081	DQ411526	AY745701	DQ408132	DQ029196
*Trichaptum byssogenum*	Dai 15555	OQ449401	OQ449085	OQ449026		OQ874771
*Trichaptum perrottetii*	JV 1908/ 45	OQ449405	OQ449092	OQ449031		OQ874776
*Tsugacorticium kenaicum*	CFMR HHB17347	JN368234		JN368221		
*Tubulicrinis accedens*	Alden Dirks:ACD0414		OL756001	OL742444		
*Tubulicrinis calothrix*	LWZ 20210919-1b	ON063835	ON063704	ON063904	ON100733	
Tubulicrinis calothrix**	SFC20180822-18	OQ996193	OQ996178	OQ996208	OR360746	
*Tubulicrinis glebulosus*	LWZ 20180903-13	ON063836	ON063705	ON063905		ON089698
*Tubulicrinis gracillimus*	HHB-13180-sp.	AF518592		AF518661		
*Tubulicrinis* sp.	SFC20180919-05		MK992866			
*Tubulicrinis subulatus*	LWZ 20190914-7	ON063837	ON063706	ON063906		
*Tubulicrinis xantha*	CLZhao 2869		MT153875	MT153882		
*Tubulicrinis yunnanensis*	CLZhao 2633		MT153877	MT153884		
*Xylodon serpentiformis*	LWZ 20190816-12a	ON063813	ON063683	ON063883	ON100712	

Bolded=  type

^*^ = new species

^**^ = unreported species

__ = newly generated sequence

### Phylogenetic Analyses

The newly generated sequences were aligned for each genetic region using MAFFT version 7 (Katoh & Standley, 2013) with reference sequences from GenBank, including those derived from specimens in Korea and other type-derived or published *Hymenochaetales* sequences (Table 1). Manual trimming was performed at the ends of the alignments. Through a preliminary maximum likelihood (ML) phylogenetic tree inference for each genetic marker, any sequence regarded as non-*Hymenochaetales* (e.g., MK269149 & OL462828) was removed for the final tree inference. The five genetic regions were concatenated using Geneious Prime 2023.0.3.

ML and Bayesian inference (BI) trees were constructed using the concatenated dataset, and *Fomitopsis pinicola* AFTOL 770, *Heterobasidion annosum* 06129/6, *Polyporus squamosus* Cui 10595, and *Tremellodendron pallidum* AFTOL 699 were used as outgroup (Wang et al., 2023). A nucleotide substitution model for each genetic marker was estimated and employed by respective phylogeny tools. The ML tree was inferred using RAxML v. 8.2.12 (Stamatakis, 2014) with 1,000 replications, and the BI tree was constructed with MrBayes v.3.2.6 (Huelsenbeck & Ronquist, 2001), starting from random trees. BI trees were sampled every 500th generation from one million generations. A 75% support majority rule consensus tree was constructed after removing the first 5% of the trees, and the Bayesian Posterior Probabilities (BPP) were calculated from the remaining trees. Species identification for each specimen was verified based on the final phylogenetic tree.

The same inference tools were used to construct *Rickenella* phylogenetic trees using a concatenated ITS and nLSU dataset. Sequences of all *Rickenella* specimens were retrieved (2023-10-14) from GenBank as a reference. Outgroup sequences selected were *Cantharellopsis prescotii* ANT285-HRL2135 and H6059300 and *Cotylidia fibrae* BJTC FM639 based on their close phylogenetic relationship with *Rickenella*, as verified in Zhang et al. (2018).

### Morphological Analyses

Morphological characters were analyzed for the studied fungarium specimens. Descriptions of macromorphological characters, such as the color, texture, and size of basidiomes, were based on field notes and photographs. For the micromorphological characters, the pileus, hymenophore, and stipe tissues were observed. The tissues were mounted in 5% KOH and stained in 1% Congo red dye. Microscopic features such as basidiospores, basidia, cystidia, and hyphae were observed under a Nikon 80i compound light microscope (Nikon) at 100 × to 400 × magnification.

For the measurements of microscopic features, at least 20 elements of basidia, basidiospores, and cystidia were selected, where possible. The 5% extreme values from each end are given in parentheses. The abbreviation ‘L’ and ‘W’ refer to the mean basidiospore length and width, respectively, ‘Q’ refers to the average length-to-width ratio of basidiospores, and [n/m/p] refers to measurements of n basidiospores from m basidiomes of p collections. Cyanophilic and iodine reactions of basidiospores were tested using Cotton Blue and Melzer’s reagent. All color descriptions follow the codes in the *Methuen Handbook of Colours* (Kornerup & Wanscher, 1978).

## Results

### Phylogenetic Analyses

**Fig. 1:**
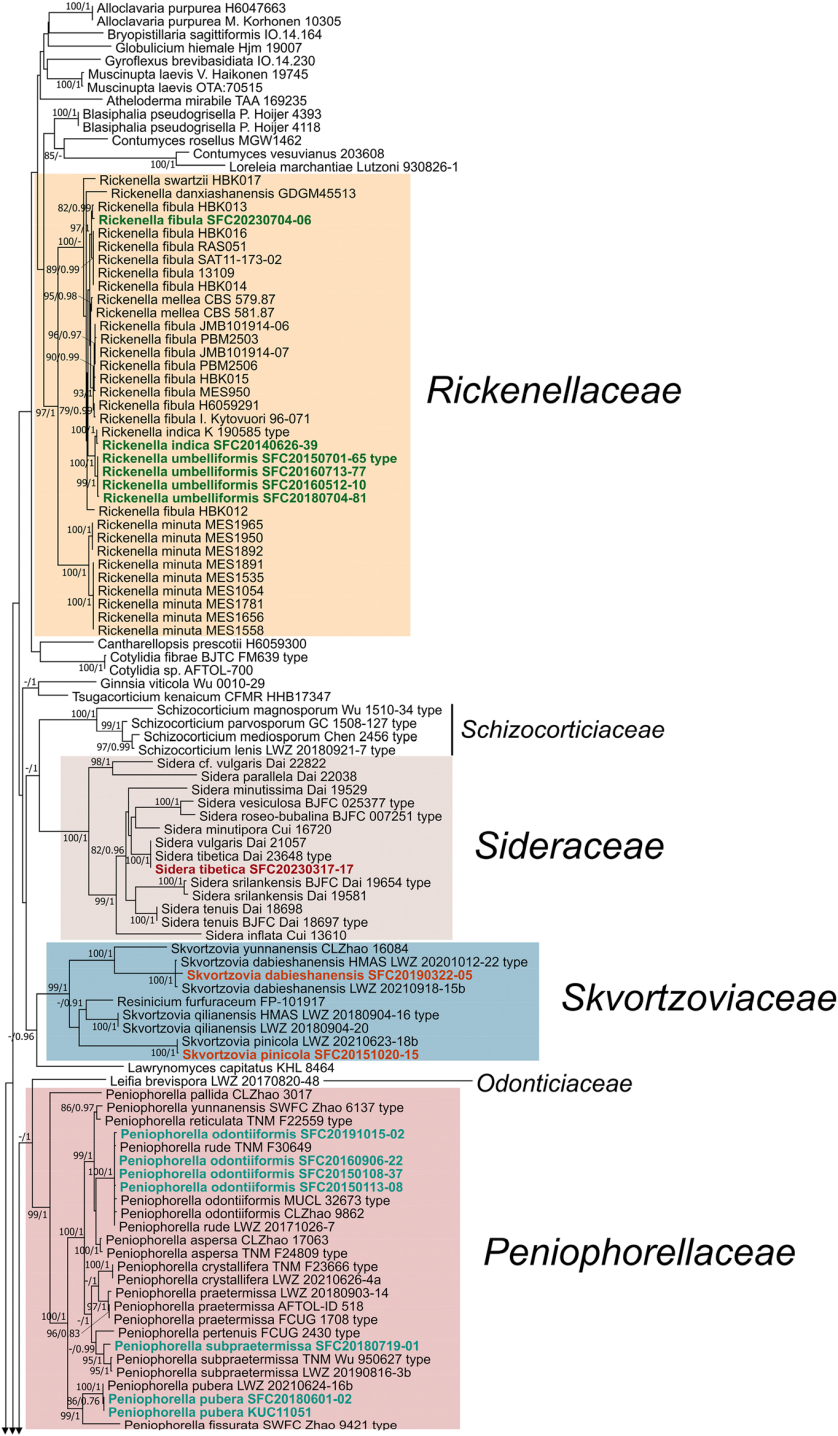

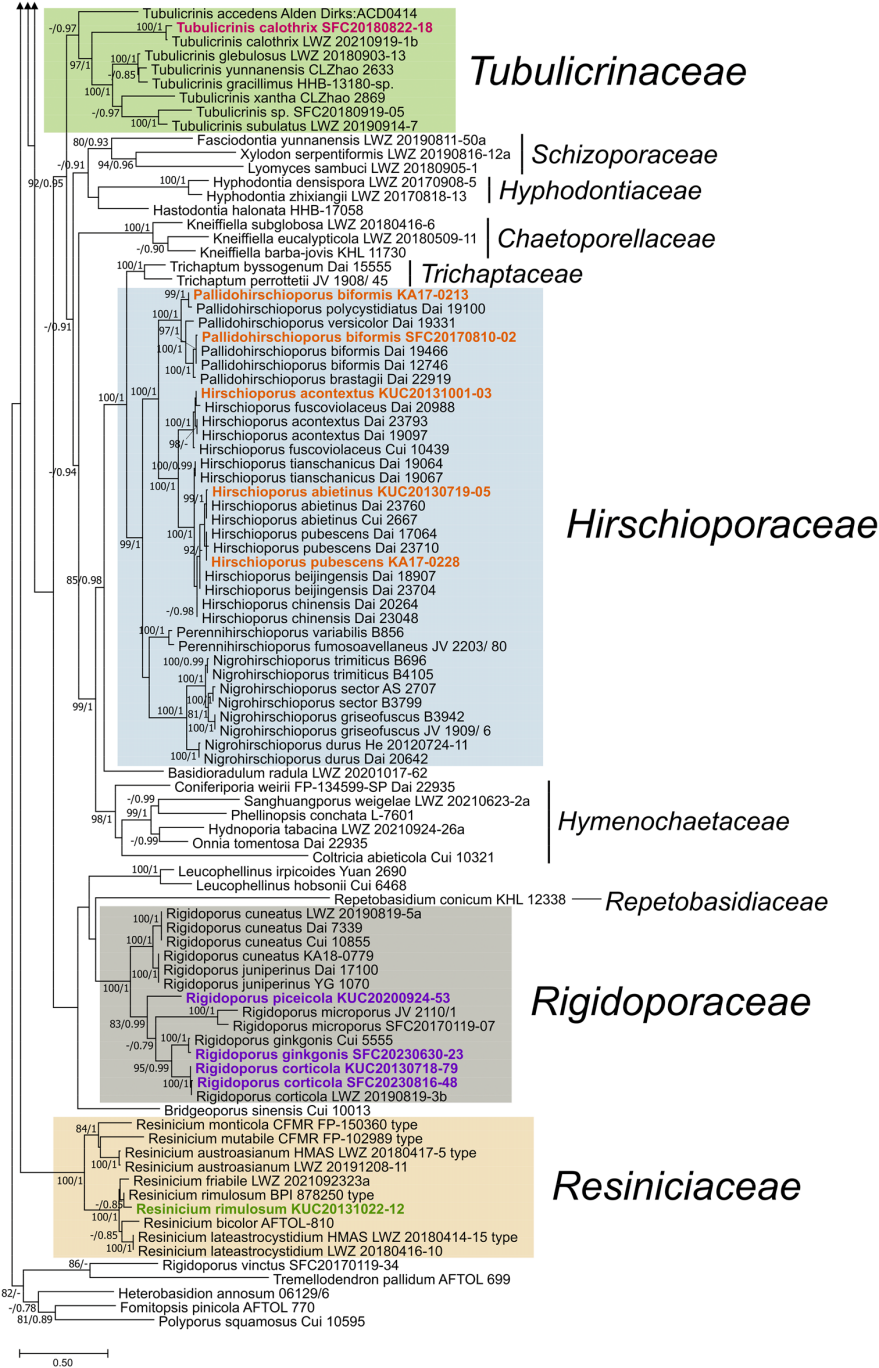
Phylogeny of *Hymenochaetales*. The phylogenetic tree was inferred using ML and BI methods (tree topology from ML) based on concatenated nSSU + ITS + nLSU + *RPB2* + *TEF1* dataset. Statistical values (ML/BI) above 75/0.75 are designated on or below each branch. Strains not indicated by any family are classified under *incertae sedis.* Colored boxes feature families with species present in Korea, and colored specimens indicate those from Korea

Three to five genetic marker sequences were newly acquired from 15 specimens. The multigenetic marker phylogenetic analysis was conducted using the concatenated alignments of 202 specimen data with 5788 bases, including gaps (Supplementary data 1): nSSU = 1087 bases; ITS = 1499 bases (ITS 1 = 821 bases, 5.8S = 160 bases, ITS2 = 518 bases); nLSU = 1412 bases; *RPB2* = 1193 bases (exon 1 = 1106 bases, intron 1 = 62 bases, exon 2 = 25 bases); *TEF1* = 597 bases (exon 1 = 150 bases, intron 1 = 85 bases, exon 2 = 107 bases, intron 2 = 89 bases, exon 3 = 166 bases). The BI phylogenetic tree (Fig. S1) topology conformed with the ML tree, supporting all 16 families as independent (Fig. 1).

**Fig. 2:**
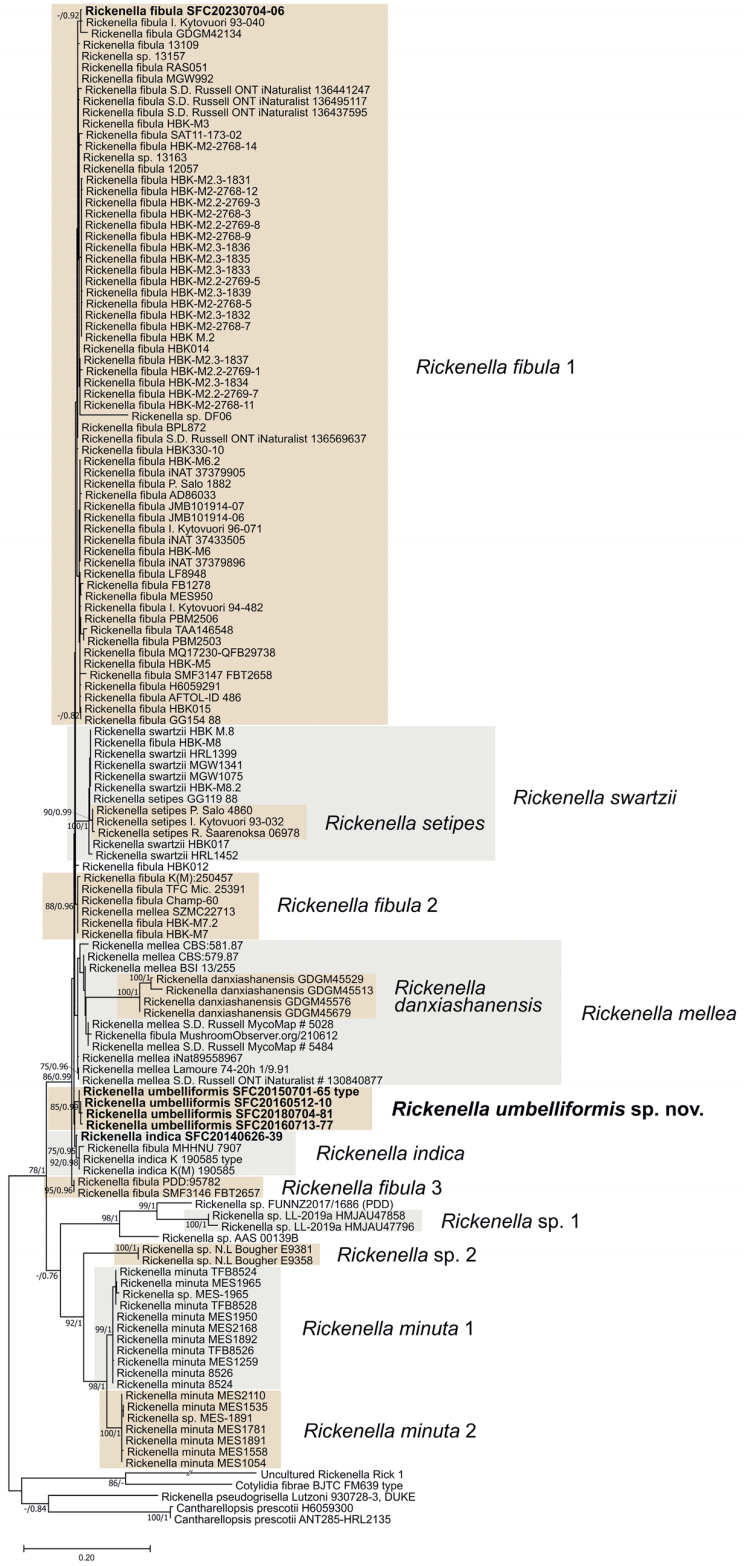
Phylogeny of *Rickenella*. The phylogenetic tree was inferred using ML and BI methods (tree topology from ML) based on concatenated ITS + nLSU dataset. Statistical values (ML/BI) above 75/0.75 are designated on or below each branch. Specimens from Korea are in bold

Exclusive of *Hymenochaetaceae* and *Schizoporaceae*, 18 species in eight understudied *Hymenochaetales* families were phylogenetically confirmed to be present in Korea (Fig. 1). Five unreported species were as follows: *Rickenella indica* in *Rickenellaceae*, *Rigidoporus ginkgonis* in *Rigidoporaceae*, *Sidera tibetica* in *Sideraceae*, *Skvortzovia dabieshanensis* in *Skvortzoviaceae*, and *Tubulicrinis calothrix* in *Tubulicrinaceae*. One species in the *Rickenellaceae* was phylogenetically revealed to be new to science. The new species, introduced here as *Rickenella umbelliformis*, was strongly supported (ML/BI support values = 99/1 in Fig. 1), even with two genetic rDNA markers (ITS and nLSU) dataset (85/0.99 in Fig. 2). The remaining 12 indigenous species in Korea were *Hirschioporus abietinus*, *H. acontextus*, *H. pubescens*, and *Pallidohirschioporus biformis* in *Hirschioporaceae*, *Peniophorella odontiiformis*, *P. pubera*, and *P. subpraetermissa* in *Peniophorellaceae*, *Resinicium rimulosum* in *Resiniciaceae*, *Rickenella fibula* in *Rickenellaceae*, *Rigidoporus corticola* and *R. piceicola* in *Rigidoporaceae*, and *Skvortzovia pinicola* in *Skvortzoviaceae.*

### Morphological Characters of Native Specimens

A combination of micromorphological characters of *Rickenella umbelliformis* sp. nov. was distinct from its closely related species, supporting its novelty. The morphology of five nationally unreported species was generally uniform with descriptions of each respective type or a widely accepted specimen. However, some differences were found in the measurements of microscopic features, such as basidiospores and basidia. Detailed comparisons are listed in the Notes sections of Taxonomy.

### Taxonomy

This section includes species descriptions of one new species and five previously unreported species from Korea.

***Rickenellaceae*** Vizzini

***Rickenella*** Raithelh.

**Fig. 3:**
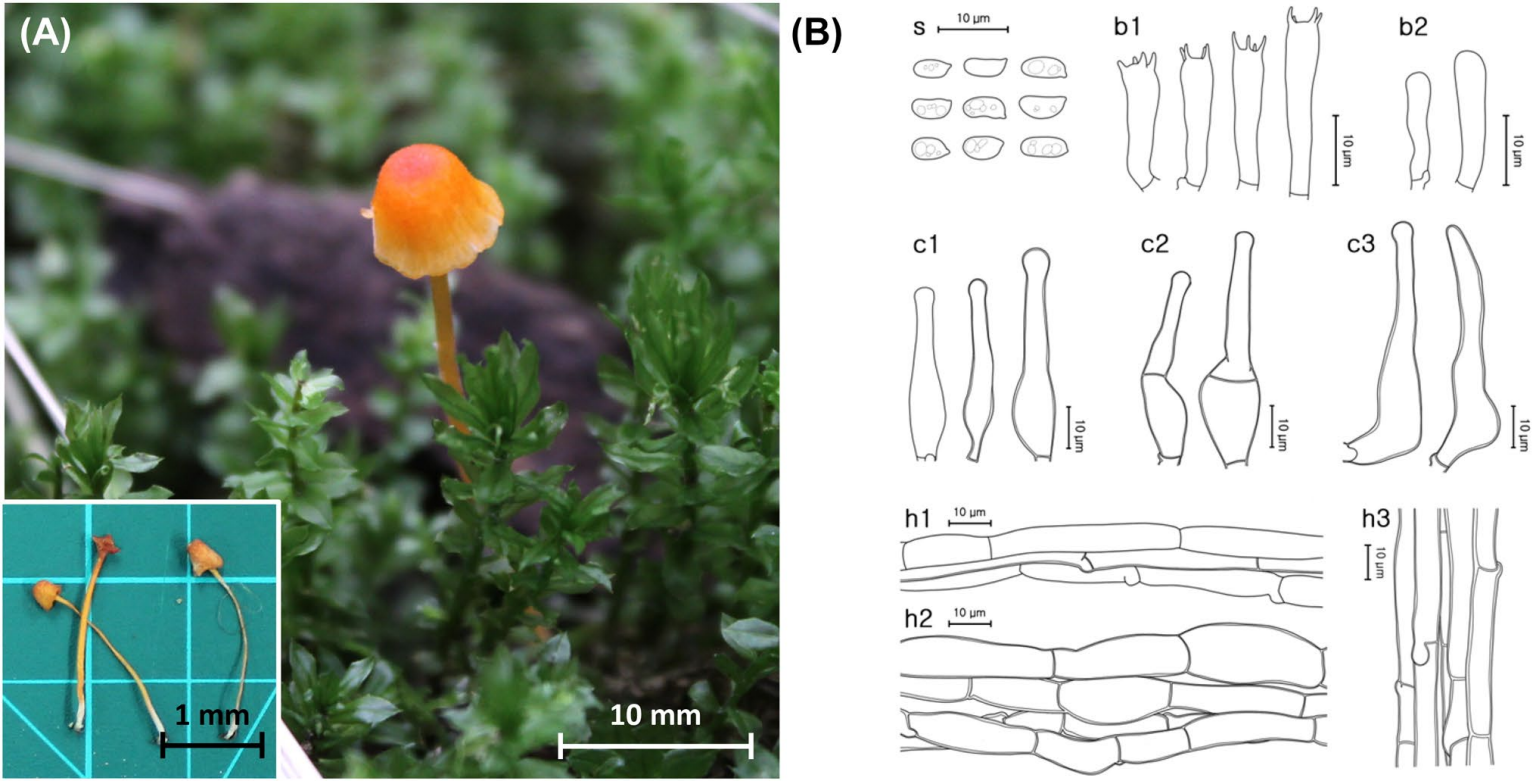
*Rickenella indica* from the Republic of Korea. **A** Basidiomes of SFC20140626-39. **B** Micromorphological characters, where ‘s’ refers to basidiospores, ‘b1’ for basidia, ‘b2’ for basidioles, ‘c1’ for cheilocystidia and pleurocystidia, ‘c2’ for pileocystidia, ‘c3’ for caulocystidia, ‘h1’ for hymenial hyphae, ‘h2’ for pileipellis hyphae, and ‘h3’ for stipitipellis hyphae

***Rickenella indica*** K. P. D. Latha & Manim., Fig. 3

**MycoBank**: MB 807702

**Description**: **Basidiomes** small, omphalinoid. **Pileus** 1.0–7.3 mm wide, downy, hygrophanous, paraboloid to convex with a shallow central depression, margin wavy, decurved with age, center reddish orange (9B8), surface yellowish orange (7A8), bleaching towards margin. **Stipe** 8.0–46.3 × 0.5–1.7 mm, central, cylindrical, downy, gelatinous, brownish yellow (5B7), gradually becomes lighter in color to the base, white at the base.

**Basidiospores** (5.6–)6.0–6.7(–7.2) × 2.8–3.4(–3.6) µm, L = 6.6 µm, W = 3.1 µm, Q = 1.8–2.2, subcylindrical, thin-walled, smooth, guttules present, cyanophilous, and neither amyloid nor dextrinoid. **Basidia** (15.5–)16.2–20.4(–20.6) × (4.3–)4.7–5.1 µm, thin-walled, clavate to narrowly utriform, 4-spored. **Cheilocystidia and pleurocystidia** indistinguishable, 38.8–53.8(–54.6) × (6.5–)7.0–12.0 µm, abundant, narrowly lageniform and obclavate, thin-walled, hyaline. **Pileus trama** hyphae septate, subregular, cylindrical or subfusoid, 13.3 µm wide on average, moderately thick-walled, hyaline, inamyloid. **Pileocystidia** 51.9–66.9 × 10.8–16.6 µm, narrowly lageniform to tibiiform, some septate, moderately thick-walled. **Stipitipellis** hyphae septate, moderately thick-walled, hyaline, inamyloid. **Caulocystidia** similar to cheilocystidia and pleurocystidia in all aspects. **Clamp connections** present.

**Ecology/Substrate/Host**: Grows on moss bed on ground.

**Distribution**: India, Republic of Korea

**Specimens examined**: Republic of Korea: Gyeonggi-do, Guri-si, Donggu-dong, Donggureung, deciduous forest, on ground moss bed of *Plagiomnium acutum*; 26 June 2014, Young Woon Lim (SFC20140626-39). Republic of Korea: Seoul, Seongdong-gu, Seoulsup 2-gil; 37°32′44″N 127°2′19″E, 16 m, Seoul forest park, mixed trees, on ground moss bed of *Trachycystis microphylla*; 02 July 2023, Shinnam Yoo (SFC20230702-01).

**Notes**: *Rickenella indica* from Korea largely resembles the holotype from India morphologically with overlapping ranges of the pileus, basidia, and pleurocystidia in size and also, ecologically, sharing the same lineage of symbiotic moss (*Bryopsida*). These biogeographically discrete specimens are also supported as the same species in the multigenetic marker phylogeny. However, they are different in several other morphological characters. *Rickenella indica* from Korea has longer and thicker stipe, more cylindrical basidiospores, and larger pileocystidia than those of the holotype with stipe 3–22 × 0.5–1 µm, basidiospores Q = 1.14–2.16, and pileocystidia similar to cheilocystidia (Latha et al., 2015).

**Fig. 4:**
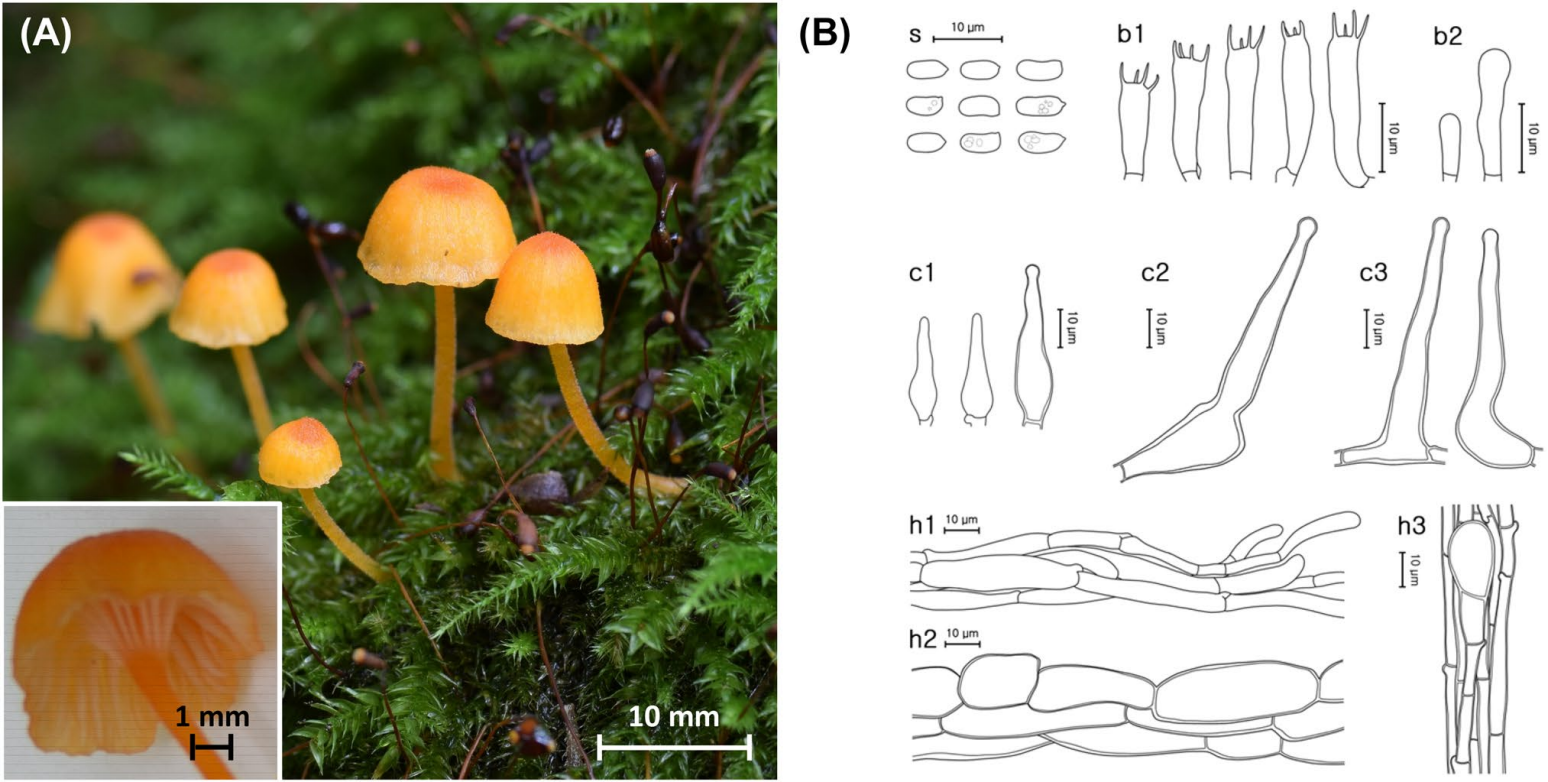
*Rickenella umbelliformis* sp. nov. **A** Basidiomes of SFC20150701-65 (holotype). **B** Micromorphological characters, where ‘s’ refers to basidiospores, ‘b1’ for basidia, ‘b2’ for basidioles, ‘c1’ for cheilocystidia and pleurocystidia, ‘c2’ for pileocystidia, ‘c3’ for caulocystidia, ‘h1’ for hymenial hyphae, ‘h2’ for pileipellis hyphae, and ‘h3’ for stipitipellis hyphae

***Rickenella umbelliformis*** Y. Cho & Y.W. Lim, sp. nov. Fig. 4

MycoBank: MB 849306

**Etymology**: ‘umbelliformis’ refers to the umbrella shape of the mushroom in Latin.

**Holotype**: Republic of Korea: Jeju-do, Jeju-si, Jocheon-eup; Jeju Provincial Park entrance Gotjawal, mixed forest, on moss bed of *Brachythecium populeum*; 1 July 2015, Young Woon Lim (SFC20150701-65, dried specimen).

**Description**: **Basidiomes** small, omphalinoid. **Pileus** 5–15 mm wide, hygrophanous, downy, paraboloid to hemispherical, slightly campanulate when young, forming central depression and wavy decurved margin with age, plicate, center reddish orange (10B8), surface yellowish orange (6A8), bleaching towards margin. **Lamellae** 12–16, deeply decurrent, white, single lamellula present between two lamellae, edge entire. **Stipe** 21–42 × 1.2–1.7 mm, central, cylindrical, downy, gelatinous, yellow (4A8) when young, yellowish orange (6A8) when mature, gradually becomes lighter in color to the base, white at the base.

**Basidiospores** [20/3/2] (4.9–)5.3–6.1(–6.5) × 2.4–2.9(–3.2) µm, L = 5.5 µm, W = 2.8 µm, Q = 1.7–2.2, subcylindrical, thin-walled, smooth, guttules present, cyanophilous, and neither amyloid nor dextrinoid. **Basidia** (15.9–)16.4–20.0 × (3.3–)3.5–5.4(–5.8) µm, thin-walled, narrowly utriform, 4-spored, sterigmata up to 3.6 µm. **Lamellar trama** hyphae septate, subregular, cylindrical or subfusoid, moderately thick-walled, hyaline, inamyloid. **Cheilocystidia and pleurocystidia** indistinguishable, (21.9–)31.6–43.3(–48.0) × (6.1–)6.3–8.9(–9.6) µm, either rare or very frequent, narrowly lageniform and sub-capitate with apex of 2.3–3.6 µm in width, thin-walled, hyaline. **Pileipellis** hyphae septate, subregular, cylindrical or subfusoid, up to 18.8 µm in width, moderately thick-walled, hyaline, inamyloid, longer than tramal hyphae. **Pileocystidia** 39.0–48.0(–60.8) × (8.4–)11.4–16.1(–17.8) µm, narrowly lageniform and sub-capitate, moderately thick-walled but thinning towards the apex of 3.0–5.5 µm in width. **Stipitipellis** hyphae septate, moderately thick-walled, hyaline, inamyloid. **Caulocystidia** (45.0–)48.2–73.2(– 95.6) × (9.2–)9.5–23.6(– 25.7) µm, narrowly lageniform and sub-capitate, moderately thick-walled but thinning towards the apex of 2.7–4.1 µm in width. **Clamp connections** observed in lamellar trama only.

**Ecology/Substrate/Host**: Grows on various Bryophyta moss beds on ground.

**Distribution**: The Republic of Korea

**Additional specimens examined**: Republic of Korea: Gyeonggi-do, Guri-si, Donggu-dong, Donggureung, deciduous forest, on ground moss bed of *Plagiomnium acutum*; 12 May 2016, Hae Jin Cho, Seobihn Lee, Vladimir Li (SFC20160512-10). Republic of Korea: Gyeongsangbuk-do, Ulleung-gun, Ulleung-eup, deciduous forest, on ground moss bed of *Trachycystis microphylla*; 13 July 2016, Jae young Park, Myung Soo Park, Nam Kyu Kim (SFC20160713-77). Republic of Korea: Jeollanam-do, Wando-gun, Gunoe-myeon, Chopyeong 1-gil, Wando Arboretum, mixed forest, on ground moss bed of *Trachycystis microphylla*; 4 July 2018, Hae Jin Cho, Ki Hyung Park (SFC20180704-81). Republic of Korea: Seoul, Gwanak-gu, Gwanak-ro; 37°27′34″N 126°56′53″E, 87 m, on ground moss bed of *Brachythecium populeum*; 12 June 2023, Yoonhee Cho, Dohye Kim, Minseo Cho (SFC20230612-01).

**Notes**: *Rickenella umbelliformis* sp. nov. either had very few or abundant cystidia based on different individual fruiting bodies. Considering that juvenile specimens often had small immature cheilocystidia and pleurocystidia, the number of cystidia may be dependent on the age and the section of the fruiting body. *Rickenella umbelliformis* is phylogenetically closely related to *R. indica*. The two species differ in many morphological features, including the size of the pileus and pleurocystidia. Compared to the new species, *Rickenella indica* has a smaller pileus (1–7 mm) and longer pleurocystidia of 31–53 µm (Latha et al., 2015). The omphalinoid shape and the size of the fruiting body of *R. umbelliformis* are similar to those of a globally common *Rickenella* species, *R. fibula* (Bull.) Raithelh., but these species are well-divided by rDNA marker-based phylogenetic analyses. Micromorphological characteristics of *R. fibula* vary widely based on its geographical location, making it difficult to make a definite comparison.

***Rigidoporaceae*** Jülich

***Rigidoporus*** Murrill

**Fig. 5:**
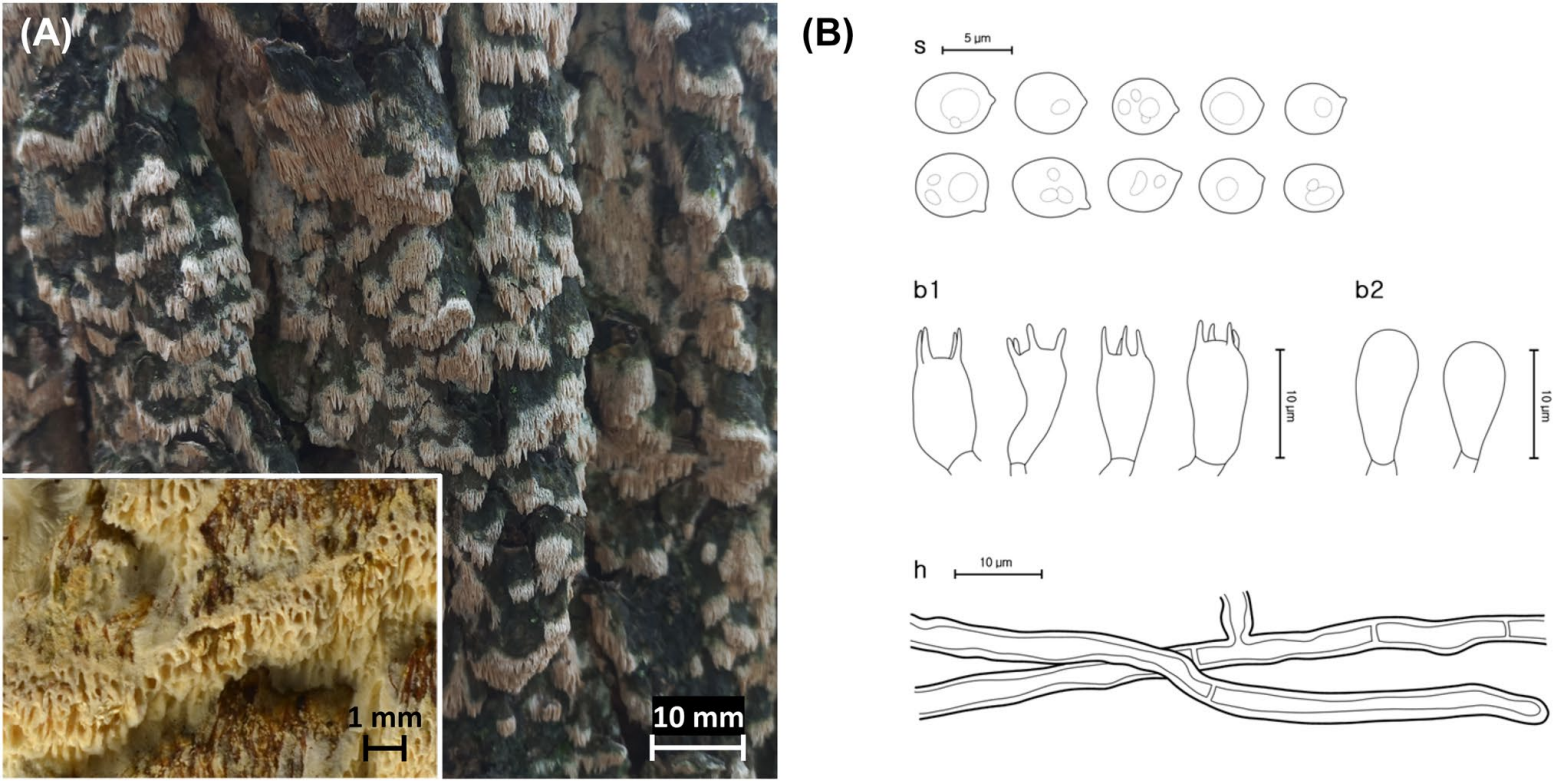
*Rigidoporus ginkgonis* from the Republic of Korea. **A** Basidiome of SFC20230630-23. **B** Micromorphological characters, where ‘s’ refers to basidiospores, ‘b1’ for basidia, ‘b2’ for basidioles, and ‘h’ for generative hyphae

***Rigidoporus ginkgonis*** (Y.C. Dai) F. Wu, Jia J. Chen & Y.C. Dai, Fig. 5

MycoBank: MB 819205

**Description**: **Basidiomes** annual, resupinate, soft corky. **Hymenophore** porous, cream (4A4), fading to white to the margin; sterile margin indistinct, thinning out; pores round or angular, irregular, 4–5 per mm; dissepiments somewhat thin, entire; tubes up to 4.0 mm deep, concolorous with the pore surface.

**Hyphal structure** monomitic, generative hyphae thin-walled, hyaline. **Basidiospores** (4.0–)4.2–5.9 × 3.4–4.4(– 4.6) μm, L = 5.0 μm, W = 4.0 μm, Q = 1.1–1.4, globose to subglobose, thin-walled, hyaline, smooth, acyanophilous, and neither amyloid nor dextrinoid. **Basidia** 10.5–13.0(– 15.0) × 4.7–6.1 μm, broadly clavate to barrel-shaped, hyaline, 4-spored. **Cystidia** absent. **Clamp connections** absent.

**Ecology/Substrate/Host**: Grows on the trunk of living or dead *Ginkgo biloba.*

**Distribution**: China, Republic of Korea, and Uzbekistan

**Specimen examined**: Republic of Korea: Gangwon-do, Yanggu, Yanggu-eup, Godae-ri; 38°07′41.6″N, 127°59′24.7″E 181 m, sidewalk of Paro-ho, on the trunk of *Ginkgo biloba*; 30 June 2023, Young Woon Lim, Hannah Suh, Dohye Kim, Yoongil Lee (SFC20230630-23).

**Notes**: Morphological characters of *Rigidoporus ginkgonis* specimens from Korea match well with the morphological descriptions of the holotype. Only a few deviations exist, where the length of the tubes is shorter for the specimens from Korea (up to 4.5 mm for the holotype), and the size of the basidiospores is smaller compared to that of the holotype with (4.8–)5.0–6.0(– 6.5) × (3.9–)4.1–5.0(– 5.2) μm, L = 5.4 μm, W = 4.5 μm (Dai & Wang, 2005).

***Sideraceae*** L.W. Zhou & Xue W. Wang.

***Sidera*** Miettinen & K.H. Larss.

**Fig. 6:**
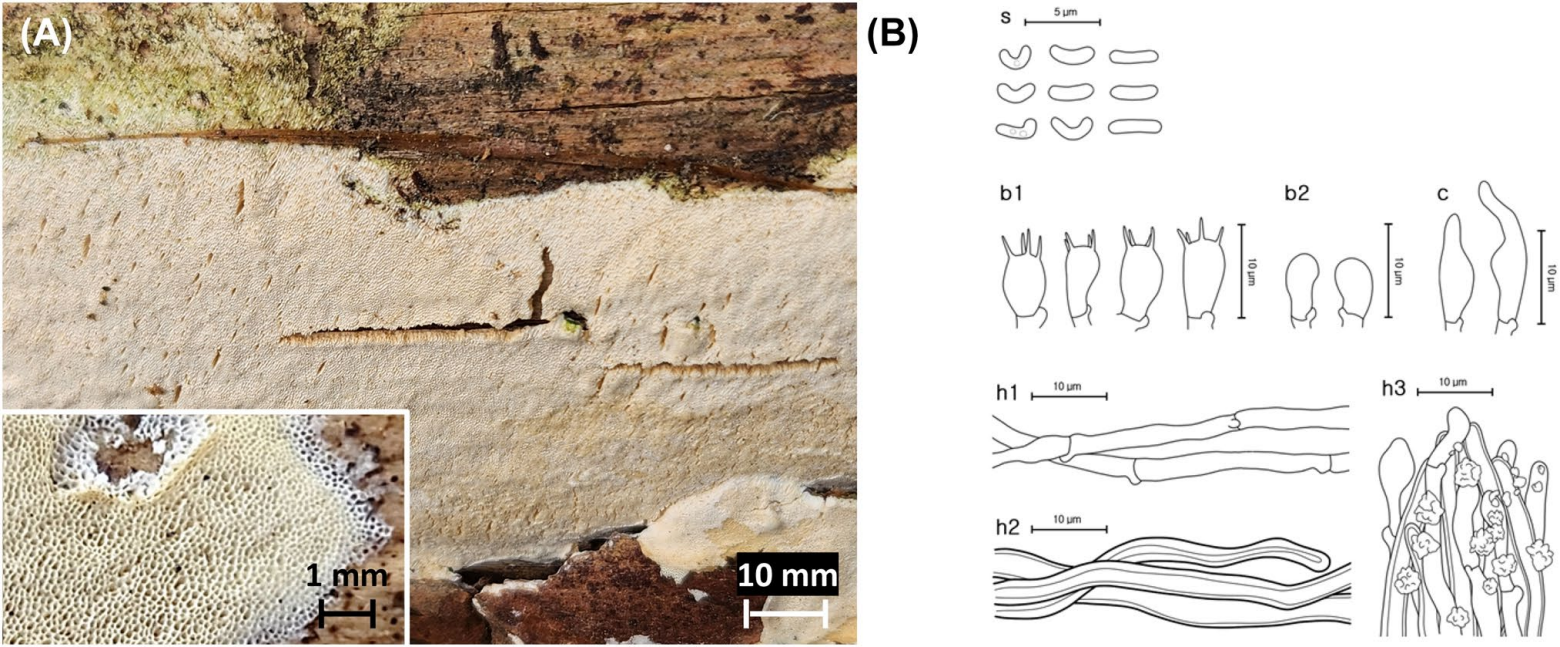
*Sidera tibetica* from the Republic of Korea**.** **A** Basidiome of SFC20230317-17. **B** Micromorphological characters, where ‘s’ refers to basidiospores, ‘b1’ for basidia, ‘b2’ for basidioles, ‘c’ for cystidioles, ‘h1’ for generative hyphae, ‘h2’ for skeletal hyphae, and ‘h3’ for crystallite hyphae

***Sidera tibetica*** Z.B. Liu, Jian Yu & F. Wu, Fig. 6

MycoBank: MB 843516

**Description**: **Basidiomes** annual, resupinate, soft corky. **Hymenophore** porous, cream (4A3), fading to white to the margin; sterile margin indistinct, thinning out; pores round but somewhat angular, 8–9 per mm; dissepiments thin, entire to lacerate; tube concolorous with the pore surface.

**Hyphal structure** dimitic, skeletal hyphae dominant, thin-walled, hyaline, rarely branched, rosette-like crystals frequently present. **Basidiospores** (1.8–)2.0–2.6(– 2.8) × 0.5–0.9 μm, L = 2.3 μm, W = 0.7 μm, Q = 2.4–4.6, allantoid to cylindrical, thin-walled, hyaline, smooth, acyanophilous, and neither amyloid nor dextrinoid. **Basidia** (8.1–)8.6–9.6(– 10.1) × (3.5–)4.0–4.4 μm, barrel-shaped to clavate, hyaline, 4-spored. **Cystidioles** 9.4–12.7 × 2.6–3.9 μm, fusoid, basally swollen, somewhat sharp tip, infrequent, thin-walled, hyaline. **Clamp connections** present.

**Ecology/Substrate/Host**: Grows on dead *Pinus* spp.

**Distribution**: China, Republic of Korea

**Specimen examined**: Republic of Korea: Gwangju, Buk-gu, Mudeung-ro; 35°8′59″N 126°59′15″E, 360 m, Mt. Mudeung, mixed forest, on the trunk of a dead *Pinus densiflora*; 17 Mar 2023, Yoonhee Cho, Dohye Kim (SFC20230317-17).

**Notes**: Morphological characters of *Sidera tibetica* specimen from Korea largely correspond to those of the holotype. The only difference lies in the sizes of the basidiospores and cystidioles. The sizes were smaller for the specimen from Korea, where the holotype bore larger basidiospores of (2.8–)2.9–3.1(– 3.3) × 1–1.1(– 1.2) μm, and cystidioles of 13–15 × 3–4 μm (Liu et al., 2022).

***Skvortzoviaceae*** L.W. Zhou & Xue W. Wang

***Skvortzovia*** Bononi & Hjortstam

**Fig. 7:**
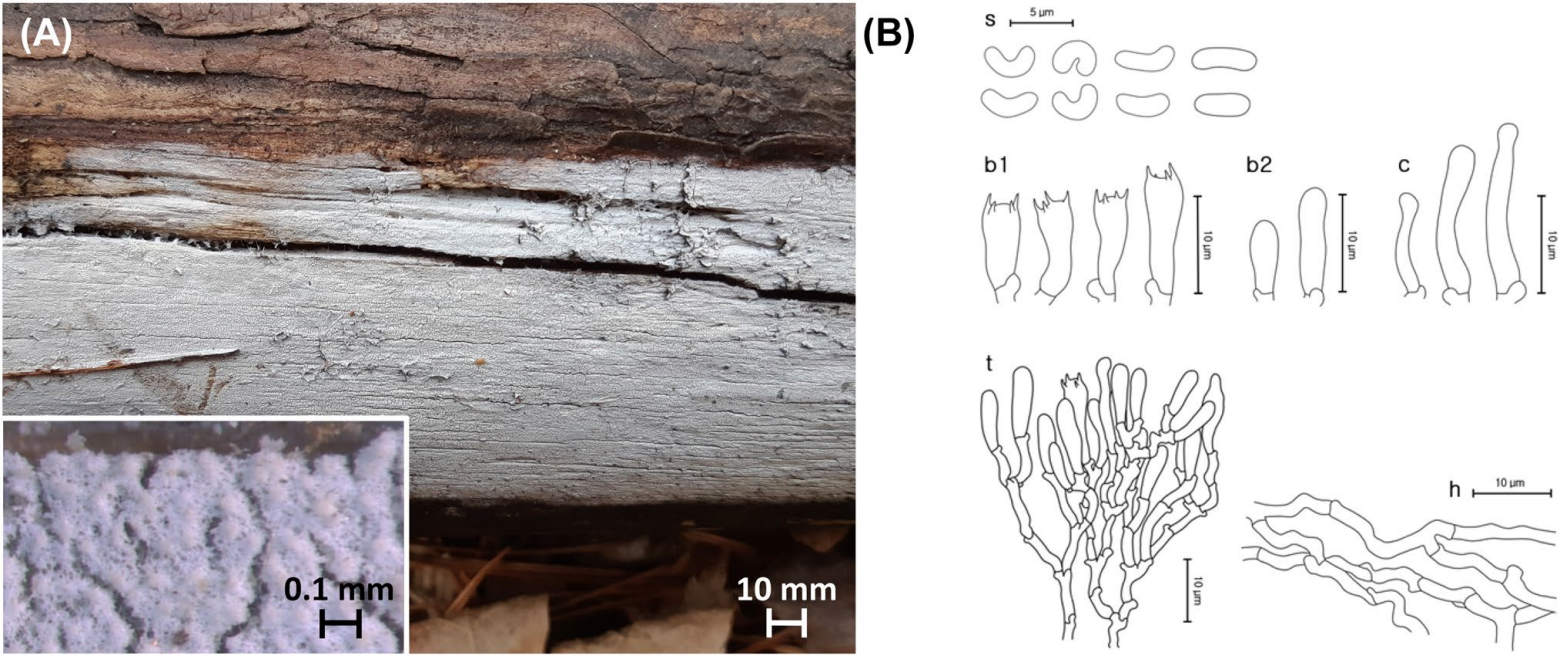
*Skvortzovia dabieshanensis* from the Republic of Korea. **A** Basidiome of SFC20190322-05. **B** Micromorphological characters, where ‘s’ refers to basidiospores, ‘b1’ for basidia, ‘b2’ for basidioles, ‘c’ for cystidioles, ‘t’ for hymenial tissue, and ‘h’ for generative hyphae

***Skvortzovia dabieshanensis*** Jia Yu, Xue W. Wang, S.L. Liu & L.W. Zhou, Fig. 7

MycoBank: MB 840228

**Description**: **Basidiomes** annual, resupinate, closely adnate, not easily separable, thin, white to ash-gray (1B1). **Hymenophore** minutely grandinoid, 9–10 aculei per mm; margin thinning out, white.

**Hyphal structure** monomitic, hyaline, thin-walled, frequently branched. **Basidiospores** 4.5–4.6 × 1.7–1.9 μm, L = 4.6 μm, W = 1.8 μm, Q = 2.4–2.6, reniform, hyaline, smooth, acyanophilous, and neither amyloid nor dextrinoid. **Basidia** 10.0–14.9(– 20.9) × 3.1–4.2 μm, narrowly obpyriform, hyaline, 4-spored. **Leptocystidia** 17.8 × 3.5 μm, cylindrical, capitate, hyaline, thin-walled. **Clamp connections** present.

**Ecology/Substrate/Host**: Grows mostly on dead *Pinus* spp.

**Distribution**: China, Republic of Korea

**Specimens examined**: Republic of Korea: Gangwon-do, Yangyang-gun, Osaek-ri; 38°4′20″N 128°26′54″E, 415 m, Mt. Jeombong, *Pinus koraiensis* forest, on the trunk of a dead *P. koraiensis*; 7 May 2016, Young Woon Lim, Jae Young Park (SFC20160907-26). Republic of Korea: Gyeongsangnam-do, Hapcheon-gun, Mt. Gaya, *Pinus densiflora* forest, on the trunk of a dead *P. densiflora*; 9 Feb 2017, Young Woon Lim, Nam Kyu Kim, Hae Jin Cho (SFC20170209-12). Republic of Korea: Gyeongsangnam-do, Hapcheon-gun, Mt. Gaya, *Pinus densiflora* forest, on the trunk of a dead *P. densiflora*; 7 Sep 2018, Hyun Lee, Nam Hwi Kim, Abel Severin Lupala (SFC20180907-152). Republic of Korea: Gangwon-do, Inje-gun, Jindong-ri, on the trunk of a dead *P. densiflora*; 29 Sep 2018, Young Woon Lim, Ki Hyeong Park, Abel Severin Lupala (SFC20180929-32). Republic of Korea: Gyeongsangbuk-do, Bonghwa-gun, Seokpo-myeon, Daehyeon-ri; 37°4′16″N 128°57′60″E, Mt. Taebaek National Park, mixed forest, on the trunk of a dead *P. densiflora*; 22 Mar 2019, Young Woon Lim, Myung Soo Park, Min-Ji Kim, Hyun Lee, Ki Hyeong Park, Shinnam Yoo, Nam Hwi Kim (SFC20190322-05).

**Notes**: Most specimens of *Skvortzovia dabieshanensis* in Yu et al. (2021) and all our observed specimens developed basidiomes on *Pinus* as a host plant. Despite the geographical separation, specimens from Korea share similar morphological characteristics with those of the holotype. Still, they are distinctive in having smaller and more frequent aculei than the holotype, which bears 5–6 aculei per mm (Yu et al., 2021). Specimens from Korea also have narrower basidiospores and broader but shorter basidia than those of the holotype with basidiospores of (3.5–)3.8–4.7(− 4.9) × 1.8–2.4(− 2.6) μm and basidia of 10–20 × 3.5–5 μm (Yu et al., 2021).

***Tubulicrinaceae*** Jülich

***Tubulicrinis*** Donk

**Fig. 8:**
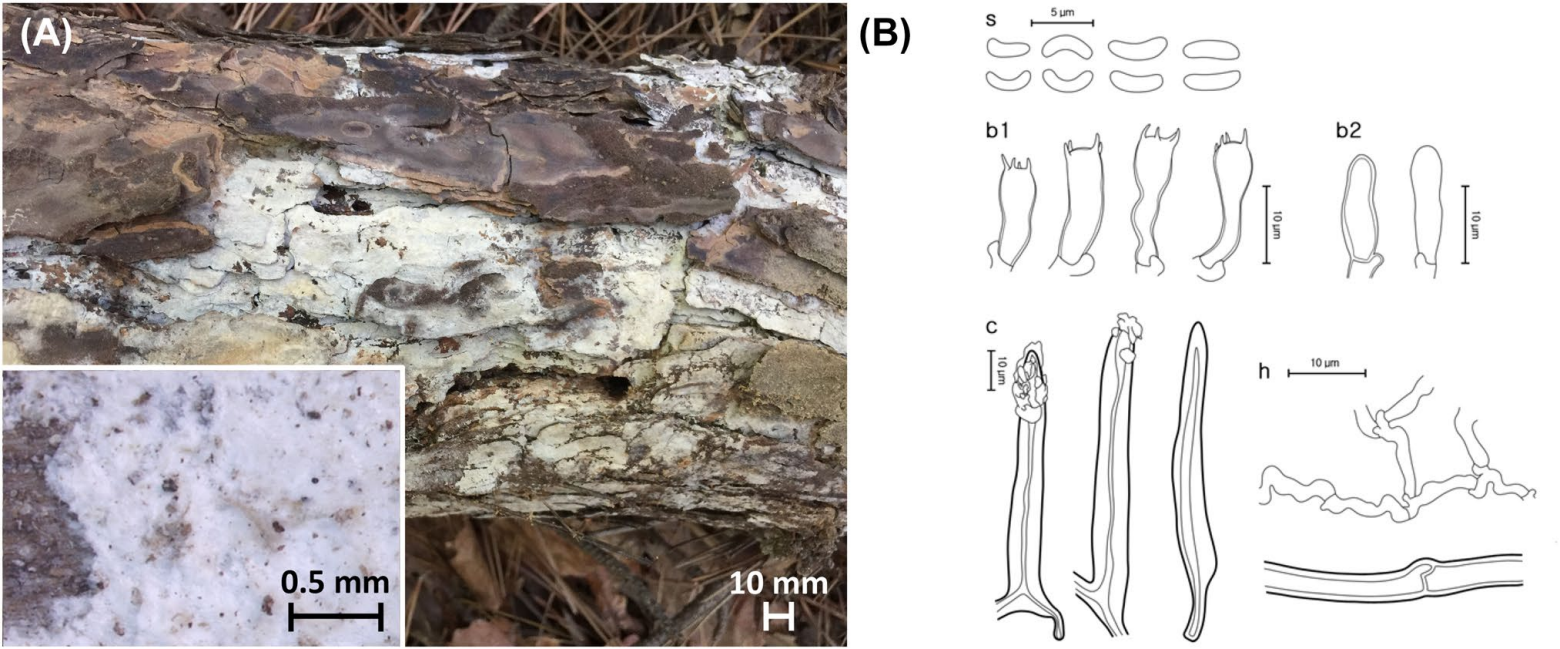
*Tubulicrinis calothrix* from the Republic of Korea. **A** Basidiome of SFC20180822-18. **B** Micromorphological characters, where ‘s’ refers to basidiospores, ‘b1’ for basidia, ‘b2’ for basidioles, ‘c’ for cystidioles, and ‘h’ for thin- and thick-walled hyphae

***Tubulicrinis calothrix*** (Pat.) Donk, Fig. 8

MycoBank: MB 307182

**Description**: **Basidiomes** annual, resupinate, adnate, thin, discontinuous, rimulose, soft. **Hymenophore** smooth, white to light brown (4A3); margin indistinct, white, thinning out.

**Hyphal structure** monomitic, hyphae thin- to thick-walled, hyaline, rarely branched in subiculum, highly branched and septate in trama. **Basidiospores** (3.3–)3.7–4.9(– 5.0) × 1.1–1.9 μm, L = 4.3 μm, W = 1.5 μm, Q = 2.3–3.7, allantoid, thin-walled, hyaline, smooth, acyanophilous, and neither amyloid nor dextrinoid. **Basidia** (9.6–)9.7–12.3(– 13.5) × (2.7–)2.9–3.6(– 3.8) μm, subclavate, hyaline, 4-spored. **Cystidia** 50.0–88.1(– 93.4) × 4.8–9.9 μm, cylindrical to setiform, numerous, hyaline, some with bent and elongated base, thick-walled but narrowing to the obtuse and sometimes encrusted apex, capillary lumen abruptly expanded near the apex, amyloid. **Clamp connections** present.

**Ecology/Substrate/Host**: Commonly found in the northern and highland; grows on coniferous wood

**Distribution**: Widely reported across all continents except for South America

**Specimen examined**: Republic of Korea: Seoul, Gwanak-gu, Mt. Gwanak, mixed forest, on the trunk of a dead *Pinus densiflora*; 22 Aug 2018, Young Woon Lim, Abel Severin Lupala (SFC20180822-18).

**Notes**: There were very few fertile cells and basidiospores in tissue where cystidia were abundant, and vice versa. The specimen from Korea largely corresponded to the morphological characters described for the specimens from Northern Europe, but several micromorphological characters were disparate. *Tubulicrinis calothrix* in Korea has shorter basidiospores, smaller basidia, and shorter cystidia than those of *T. calothrix* from Northern Europe with basidiospores of 6–7(– 8) × 1.5–1.8(– 2) μm, basidia of 12–15 × 4–5 μm, and cystidia of 80–120 × 6–8 μm (Hjortstam et al., 1988).

## Discussion

Species of *Hymenochaetales* families, apart from *Hymenochaetaceae* and *Schizoporaceae*, pose difficulties to study as they are often small, uncommon, or locality-specific (Nakasone & Burdsall, 2012), with some being endangered or threatened (Denchev et al., 2007; Karadelev & Rusevska, 2016; Tănase & Pop, 2005). Most of the understudied families were formerly placed under *Rickenellaceae*. Recently, it has been divided into several monogeneric families, i.e., *Odonticiaceae*, *Peniophorellaceae*, *Resiniciaceae*, *Schizocorticiaceae*, *Sideraceae*, and *Skvortzoviaceae*, based on multigenetic marker phylogeny and time divergence analysis (Wang et al., 2023).

The four resupinate unreported species, *Rigidoporus ginkgonis*, *Sidera tibetica*, *Skvortzovia dabieshanensis*, and *Tubulicrinis calothrix* may be easily mistaken as other species in the field as their insubstantial macromorphological characters overlap with other corticoid or polyporous species of *Hymenochaetales* and other families. In fact, before molecular work was introduced in fungal taxonomy, *Tubulicrinis calothrix* was reported as *Corticium* based on its effused basidiome, cream hymenophore, powdery margin, and abundant large cystidia (Patouillard et al., 1897). After its transfer to *Tubulicrinis* by Donk (1956), *Tubulicrinis* was only later verified through nLSU for its placement in *Hymenochaetales* (Larsson et al., 2006). Similarly, *Rigidoporus ginkgonis*, like many other *Rigidoporus* species, has been initially reported under *Oxyporus* based on morphology but has undergone a systematic transition later based on phylogeny (Wu et al., 2017). Therefore, it is incumbent for researchers to validate the classification and identification of many species with poor morphological traits through phylogenetic analyses.

Small bryophilous orange omphaloid mushrooms have been repeatedly reported as *Rickenella fibula* in Korea based on their macromorphology (Jo et al., 2013; Kim et al., 2017). However, our phylogenetic analysis presents three *Rickenella* species in the country — *R. fibula*, *R. indica*, and *R. umbelliformis* sp. nov. (Figs. 1 and 2, Fig. S2). As *Rickenella* species are challenging to identify based on morphology due to overlapping characters, phylogenetic support is essential. *Rickenella* species possess significant values for ecological and evolutionary research as they consist of a wide range of morphological characters and lifestyles, from saprotrophic to bryicolous, that may be symbiotic or parasitic to the hosts (Bresinsky & Schötz, 2006; Korotkin et al., 2018). However, prior to other research, a concrete taxonomic study is inevitable for the genus as some *Rickenella* species are not phylogenetically well resolved. The clades of *R. setipes* and *R. danxiashanensis* are nested within *R. swartzii* and *R. mellea*, respectively. *Rickenella fibula* is paraphyletic, and two new species candidates were detected, annotated as *Rickenella* sp. 1 and 2, respectively (Fig. 2).

Despite how infallible phylogenetic analysis may seem, morphological assessment is essential when differentiating phylogenetically closely related species. *Peniophorella* specimens analyzed in the present study were grouped monophyletically with both *P. odontiiformis* and *P. rude* (Fig. 1). However, they were eventually identified as *P. odontiiformis* based on basidiospore measurements (Table S1). The basidiospore measurements of the studied specimens closely matched the range of *P. odontiiformis* (7.5–9 × 3.5–5 μm) than that of *P. rude* (9–10 × 6 μm) (Larsson, 2007)*.* This problem arose partially due to some inaccurate GenBank annotations, such as DQ647494 and DQ647497, that were annotated as *P. rude* when they were identified as *P. odontiiformis* in the reference research article (Hallenberg et al., 2007). This stipulates the need to evaluate morphological characters in addition to molecular analysis for accurate species identification.

A consistent effort to update indigenous species to the most recent taxonomic classification is imperative. This is because various problems may arise from the persistent use of old names or synonyms, such as losing track of type specimens, misidentification, and inconsistent ecological and morphological descriptions of species (Dayarathne et al., 2016). *Trichaptum* has long been classified as *incertae sedis* (uncertain placement) due to its undecisive taxonomic position at the family level within *Hymenochaetales*. Recently, Zhou et al. (2023) resurrected *Trichaptum *sensu stricto to *Trichaptaceae* Y.C. Dai, Yuan Yuan & Meng Zhou, and introduced another new family, *Hirschioporaceae* Y.C. Dai, Yuan Yuan & Meng Zhou, to encompass some species in *Trichaptum *sensu lato. Based on our results, all *Trichaptum* specimens in Korea are now classified under *Hirschioporaceae* as *Hirschioporus* or *Pallidohirschioporus* species (Fig. 1). As such, there is currently no *Trichaptum* species in the country until a new discovery occurs. As *Trichaptum *sensu lato species are documented and subjected to comprehensive research across diverse research areas in Korea (Cho et al., 2020; Kim et al., 1998; Lupala et al., 2019), it is critical to contemplate taxonomic revisions to prevent potential confusion across all studies.

In conclusion, the multistage morphological and multigenetic marker-based phylogenetic investigation revealed 18 species in eight understudied *Hymenochaetales* families (exclusive of *Hymenochaetaceae* and *Schizoporaceae*) from Korea. The most recent taxonomic classification and revisions were reflected upon these species. The national species list will extend with continuous findings and research. Limited specimen records and genetic marker sequences are available for the species under *incertae sedis*. As most understudied species possess inconspicuous fruiting bodies, a more attentive fruiting body collection and genetic marker sequence production are required to expand and enrich the taxonomic studies of *Hymenochaetales*. The effort will further fill the gaps of unsupported taxa and eventually assign all genera and species to the appropriate classification.

### Supplementary Information

Below is the link to the electronic supplementary material.

Supplementary Data 1. Concatenated nSSU+ ITS+ nLSU+ RPB2+ TEF1 dataset for multigenetic marker phylogenetic analysis of Hymenochaetales

Supplementary file2 (PDF 913 KB)

Supplementary file3 (XLSX 11 KB)

## Data Availability

The study materials and data from this study are available from the corresponding author upon reasonable request.
