# Supplementary material for: Phylogenetic Assessment of Understudied Families in Hymenochaetales (Basidiomycota, Fungi)—Reporting Uncovered Species and Reflecting the Recent Taxonomic Updates in the Republic of Korea
Source: J Microbiol. 2024 May 16;62(6):429–47. doi: 10.1007/s12275-024-00120-5 (PMC11224081; doi:10.1007/s12275-024-00120-5)
Supplement: Supplementary file 2 — Supplementary file2 (PDF 913 KB) [file 12275_2024_120_MOESM2_ESM.pdf]

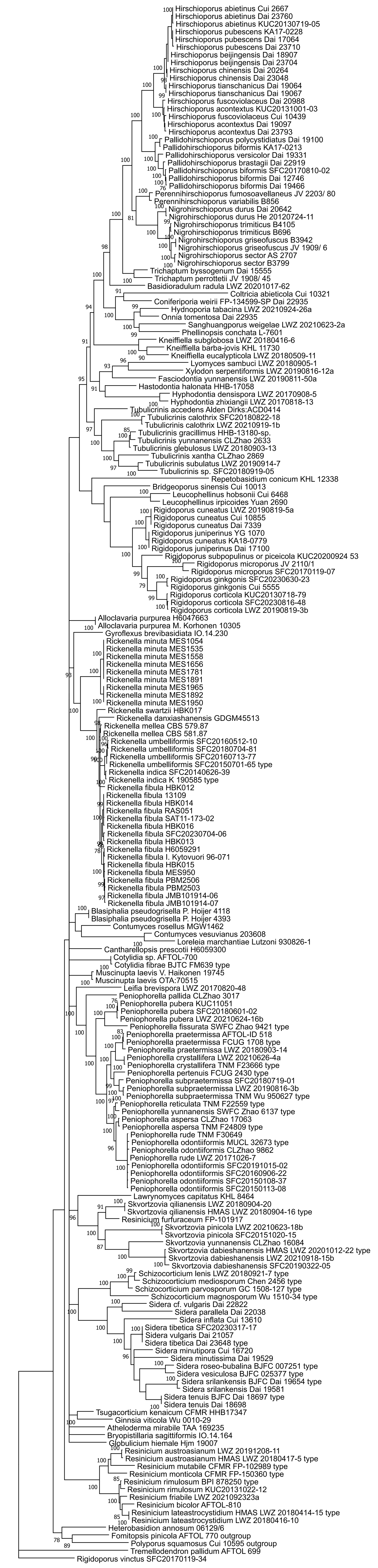

Fig. S1. BI phylogeny of *Hymenochaetales*. The BI phylogenetic tree was inferred based on concatenated nSSU+ ITS+ nLSU+ *RPB2*+ *TEF1* dataset. BPP values above 0.75 are designated on or below each branch.

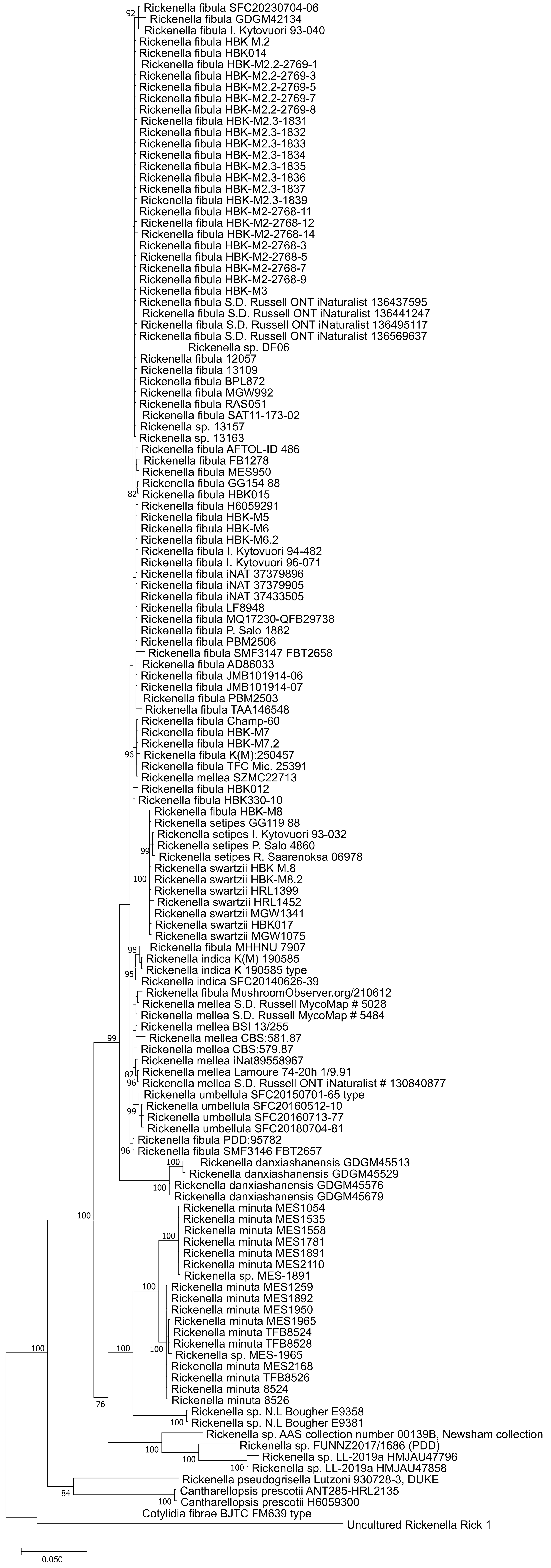

Fig. S2. BI phylogeny of *Rickenella*. The BI phylogenetic tree was inferred based on concatenated ITS+ nLSU dataset. BPP values above 0.75 are designated on or below each branch.
